# *Terminalia Chebula* provides protection against dual modes of necroptotic and apoptotic cell death upon death receptor ligation

**DOI:** 10.1038/srep25094

**Published:** 2016-04-27

**Authors:** Yoonjung Lee, Hee Sun Byun, Jeong Ho Seok, Kyeong Ah Park, Minho Won, Wonhyoung Seo, So-Ra Lee, Kidong Kang, Kyung-Cheol Sohn, Ill Young Lee, Hyeong-Geug Kim, Chang Gue Son, Han-Ming Shen, Gang Min Hur

**Affiliations:** 1 Department of Pharmacology, Research Institute for Medical Science, College of Medicine, Chungnam National University, Daejeon 301-131, Republic of Korea; 2 Department of Dermatology, College of Medicine, Chungnam National University, Daejeon 301-131, Republic of Korea; 3 Eco-Friendly New Materials Reserch Center, Korea Research Institute of Chemical Technology, 141 Daejeon 34114, Republic of Korea; 4 Liver & Immunology Research Center, Oriental Hospital of Daejeon University, Daejeon 302-122, Republic of Korea; 5 Department of Physiology, Yong Loo Lin School of Medicine, National University of Singapore, Singapore

## Abstract

Death receptor (DR) ligation elicits two different modes of cell death (necroptosis and apoptosis) depending on the cellular context. By screening a plant extract library from cells undergoing necroptosis or apoptosis, we identified a water extract of *Terminalia chebula* (WETC) as a novel and potent dual inhibitor of DR-mediated cell death. Investigation of the underlying mechanisms of its anti-necroptotic and anti-apoptotic action revealed that WETC or its constituents (e.g., gallic acid) protected against tumor necrosis factor-induced necroptosis via the suppression of TNF-induced ROS without affecting the upstream signaling events. Surprisingly, WETC also provided protection against DR-mediated apoptosis by inhibition of the caspase cascade. Furthermore, it activated the autophagy pathway via suppression of mTOR. Of the WETC constituents, punicalagin and geraniin appeared to possess the most potent anti-apoptotic and autophagy activation effect. Importantly, blockage of autophagy with pharmacological inhibitors or genetic silencing of Atg5 selectively abolished the anti-apoptotic function of WETC. These results suggest that WETC protects against dual modes of cell death upon DR ligation. Therefore, WETC might serve as a potential treatment for diseases characterized by aberrantly sensitized apoptotic or non-apoptotic signaling cascades.

Members of the tumor necrosis factor (TNF) receptor super family, which includes a group of highly conserved plasma membrane receptors with homology in their extracellular domain, exhibit variable numbers of cysteine-rich domains involved in binding their respective cognate ligands^1^. Within the TNF receptor superfamily, death receptors (DRs) such as TNF receptor I (TNFR1) and TNF-related apoptosis-inducing ligand (TRAIL) receptors are characterized by a cytoplasmic sequence termed the death domain^2^,^3^. Upon ligation, the death domain of TNFR1 engages downstream adaptor proteins and initiates the caspase-dependent apoptotic signaling cascade. During apoptotic cell death, the recruitment of Fas-associated death domain protein (FADD) and subsequent activation of initiator cysteine protease caspase-8 and caspase-10 lead to TNF-induced apoptotic cell death^4^,^5^,^6^. Although caspases play an essential role in apoptosis, they are not required for other modes of DR-mediated cell death. Indeed, a number of DRs including TNFR1, FAS, and TRAIL receptor can elicit caspase-independent cell death in some types of cells in the absence of caspase activity^7^,^8^,^9^,^10^. Such caspase-independent necroptotic cell death by DRs may involve the accumulation of reactive oxygen species (ROS). Depending on the cellular context, DRs regulate the close interplay between apoptosis and necroptosis in response to the same stimuli. Importantly, both types of cell death occur *in vivo* not only during normal physiological processes but also in pathological conditions such as myocardial ischemia, stroke, and TNF-induced inflammatory response syndrome^11^,^12^. Thus, the discovery of compounds that regulate apoptotic and necroptotic pathways will be useful for developing therapeutic approaches for relevant diseases.

During our search for regulators of DR-mediated cell death using a natural product library, the water extract of *Terminalia chebula* (WETC) was found to possess a dual inhibitory effect on both apoptosis and necroptosis. *T*. *chebula,* a member of the Combretaceae family, is a popular traditional medicine in India and East Asia^13^. It exhibits a variety of *in vivo* pharmacological activities, such as anti-aging, anti-ulcer, cardioprotection, and wound healing^14^,^15^,^16^,^17^. Moreover, it has antioxidant and free radical-scavenging activities *in vitro*^18^,^19^, which may explain its ability to protect against oxidative stress-mediated necrosis. Previous phytochemical studies have revealed that the extract of *T*. *chebula* contains geraniin, punicalagin, terflavin B, and gallic, chebulagic, chebulic, chebulinic, and tannic acids^20^,^21^. Although several studies have reported the cytoprotective effects of *T*. *chebula*, other studies on the effects of its principle constituents on cell death have been conflicting and controversial. For instance, some studies have reported that chebulagic acid, punicalagin, and geraniin induce apoptosis in several types of cancer cell, while other studies have demonstrated protection from cell death in response to DNA damage or oxidative stress^22^,^23^,^24^,^25^,^26^,^27^,^28^. Elucidating the effects of the principle constituents of *T*. *chebula* on apoptosis or necroptosis will be informative for developing potential therapeutic approaches for regulating the specific modes of cell death. Therefore, we investigated the protective effects of *T*. *chebula* and its constituents on the dual modes of cell death (apoptosis and necroptosis) induced by DR ligation, and the underlying mechanisms.

## Results

### Phytochemical characterization of WETC

**Figure 1:**
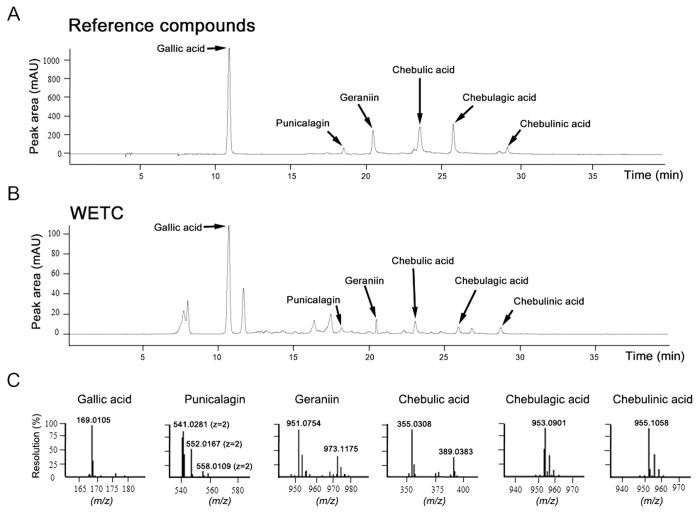
Chemical composition analysis of WETC determined using HPLC and UHPLC-HRMS analysis. HPLC chromatograms of a mixture of six reference compounds (gallic acid, punicalagin, geraniin, chebulic acid, chebulagic acid and chebulinic acid) (**A**) and WETC (**B**) monitored at 276 nm. (**C**) Mass spectra of six reference compounds by UHPLC-HRMS. Gallic acid (*m/z* 169.0105 [M-H]^−^, TIC mode); Punicalagin (*m/z* 541.0281 [M-2H]^2−^ *z* = 2, MRM mode); Geraniin (*m/z* 951.0754 [M-H]^−^, MRM mode); Chebulic acid (*m/z* 355.0308 [M-H]^−^, MRM mode); Chebulagic acid (*m/z* 953.0901 [M-H]^−^, MRM mode); Chebulinic acid (*m/z* 955.1058 [M-H]^−^, MRM mode).

To identify the chemical composition of WETC, we used high-performance liquid chromatography (HPLC) and ultra-high-performance liquid chromatography analysis coupled with tandem time-of-flight mass spectrometry (UHPLC-MS). The chromatographic data revealed the presence of gallic acid, punicalagin, geraniin, chebulic acid, chebulagic acid and chebulinic acid by comparison with the respective reference compounds (Fig. 1A,B). High-resolution mass spectra analysis further confirmed the chemical formulae of these six compounds (Fig. 1C, Supplementary Fig. 1). These results are consistent with those from previous reports^20^,^21^ on the constituents of *T*. *chebula*, although some constituents such as terflavin B and ellagic acid were not observed in our experimental conditions. Furthermore, UHPLC-MS-based quantitative analysis using the standard curve from the corresponding peak area showed that gallic acid was the most abundant constituent of WETC, followed by geraniin, chebulic acid, punicalagin, chebulinic acid, and chebulagic acid (Table 1, Supplementary Fig. 2). Quantitative results of the WETC constituents are summarized in Table 1.

### WETC provides protections against TNF-induced necroptotic cell death by suppressing ROS production

**Figure 2:**
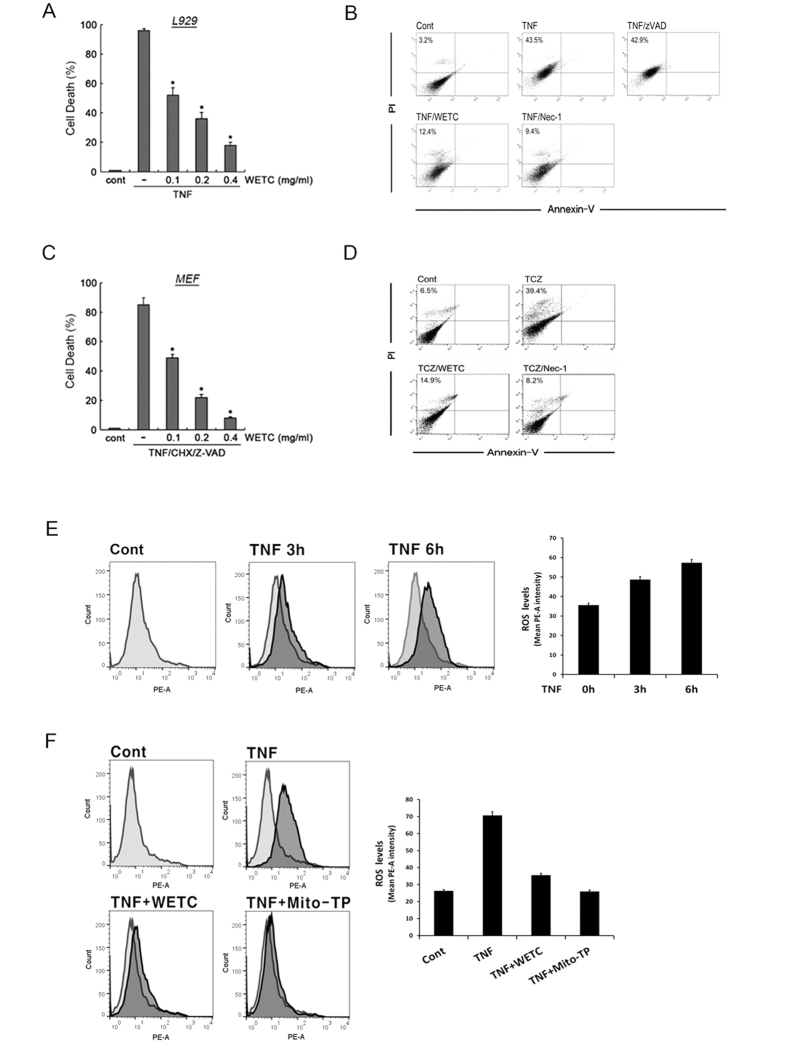
WETC protects TNF-induced necroptotic cell death by suppressing ROS production. (**A**) L929 cells were pretreated with indicated concentrations of WETC for 30 min, followed by TNF (15 ng/mL) for another 8 h. Cells were trypsinized and collected in PBS, and cell death was quantified by trypan blue exclusion assay. Data were normalized to the rate of spontaneous cell death occurring in untreated cells. Data represent the mean ± SE of three independent experiments. *P < *0.05*, compared with TNF-treated group. (**B**) L929 cells were pretreated with a pancaspase inhibitor (z-VAD-FMK, 20 μM), a necroptosis inhibitor (Nec-1, necrostatin-1, 10 μM) and WETC (0.4 mg/mL), followed by TNF (15 ng/mL) for another 8 h. The cells were stained with FITC-labeled annexin V and PI, and then analyzed by flow cytometry. (**C**) MEFs were pretreated with indicated concentrations of WETC for 30 min, and then treated with z-VAD-FMK (20 μM), TNF (15 ng/mL) and cycloheximide (CHX, 10 μg/mL) for 18 h. Cell death was quantified as in (**A**). *P < *0.05*, compared with TNF/CHX/z-VAD-FMK-treated group. (**D**) MEFs were pretreated with WETC (0.4 mg/mL) and Nec-1 (10 μM), and then treated with TNF/CHX/z-VAD-FMK as in *C*. The mode of cell death was assessed as in (**B**). (**E,F**) L929 cells were treated with TNF (15 ng/mL) in the absence or presence of WETC (0.4 mg/mL) or mitochondria-targeted antioxidant Mito-TEMPO (100 μM) for various times, as indicated. The levels of intracellular ROS were monitored using the cell permeable dye, CM-H2DCFDA as described in Methods. Left panel, representative flow cytometry data. Right panel, quantification of ROS from three independent experiments.

To identify a necroptosis inhibitor from a plant extract library, we used TNF-induced necroptosis in L929 cells, a well-established necroptotic cell death model^29^,^30^. As shown in Fig. 2A, pretreatment of L929 cells with WETC significantly suppressed TNF-induced cytotoxicity in a dose-dependent manner. To confirm that the mode of cell death by TNF was necroptosis-related, cell death was analyzed by annexin V and propidium iodide (PI) staining followed by flow cytometry. Treatment with TNF resulted in increased numbers of PI^+^ cells, whereas very few cells were exclusively stained with annexin V. (Fig. 2B, Supplementary Fig. 3A). As reported earlier^31^, pretreatment with necrostatin, a necroptosis inhibitor (but not with z-VAD-FMK, a pan-caspase inhibitor) drastically abrogated TNF-induced cell death, confirming that TNF elicits necroptotic, rather than apoptotic, cell death in L929 cells. Under TNF-induced necroptotic conditions, pretreatment of L929 cells with WETC led to a significant reduction in the PI^+^ cell population. Therefore, WETC protected against necroptotic cell death induced by TNF in L929 cells. To confirm these results, we examined the effects of WETC on mouse embryonic fibroblasts (MEFs), which undergo necroptotic cell death in response to TNF treatment in the presence of CHX and z-VAD-FMK^32^. WETC treatment drastically attenuated the cytotoxicity and diminished the PI^+^ cell population of MEFs treated with a combination of TNF, CHX, and z-VAD-FMK (Fig. 2C,D, Supplementary Fig. 3B), similar to the results found in L929 cells.

ROS are required for TNF-induced necroptotic cell death in L929 cells and MEFs^30^,^32^. As *T*. *chebula* is known for its antioxidant and free radical-scavenging activities^18^,^19^, we evaluated whether the anti-necroptotic function of WETC was due to suppression of ROS production. As expected, elevated ROS levels were detectable after TNF treatment (Fig. 2E, Supplementary Fig. 3C) and this increase was significantly attenuated when the cells were pretreated with Mito-TEMPO, a specific mitochondria-targeting antioxidant (Fig. 2F, Supplementary Fig. 3D). Similarly, pretreatment with WETC significantly suppressed TNF-induced ROS accumulation. These data indicate that WETC antagonizes mitochondrial-driven ROS production upon TNF receptor ligation, thereby inhibiting necroptosis.

**Figure 3:**
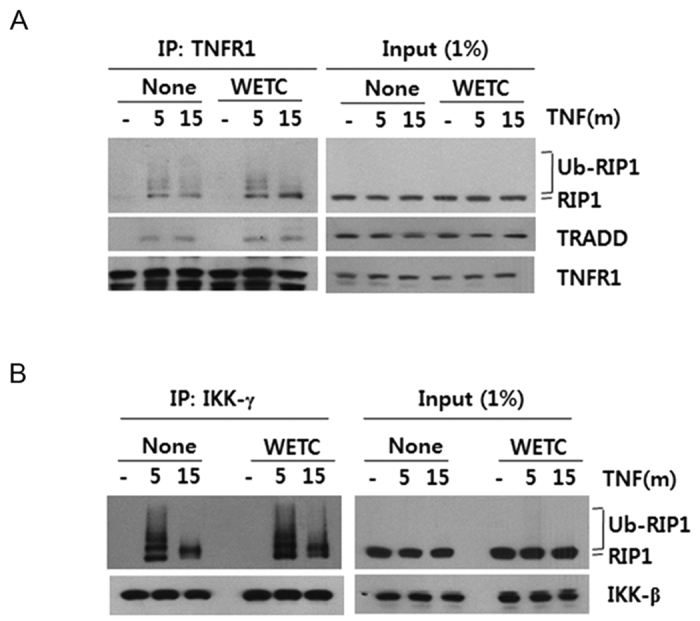
WETC does not affect the upstream signaling complex formation of TNFR1. L929 cells were treated with TNF (15 ng/mL) for 5 or 15 min in the absence or presence of WETC (0.4 mg/mL). Cell extracts from each sample were subjected to immunoprecipitation with anti-TNFR1 (**A**) and anti-IKK-γ (**B**) antibodies. Immunoprecipitates were analyzed by immunoblotting with indicated antibodies. One percent of cell extract from each treated sample was used as a control for protein content (Input).

To further assess whether WETC affects the upstream signaling events of the necroptotic pathway, we examined its effects on the formation of TNFR1 signaling complex. Notably, in the presence of WETC, TNF-induced recruitment of TRADD and hyperubiquitinated RIP1 into TNFR1 were found to be rather similar (Fig. 3A). Moreover, treatment of cells with TNF led to an immediate interaction between IKK-γ and ubiquitinated RIP1, and the extent of ubiquitinated RIP1-IKK interaction was unaffected by WETC (Fig. 3B). These results provide direct evidence that the anti-necroptotic effect of WETC is not due to inhibition at a point upstream of mitochondrial ROS.

### Protective effects of WETC constituents on DR-mediated necroptotic cell death

**Figure 4:**
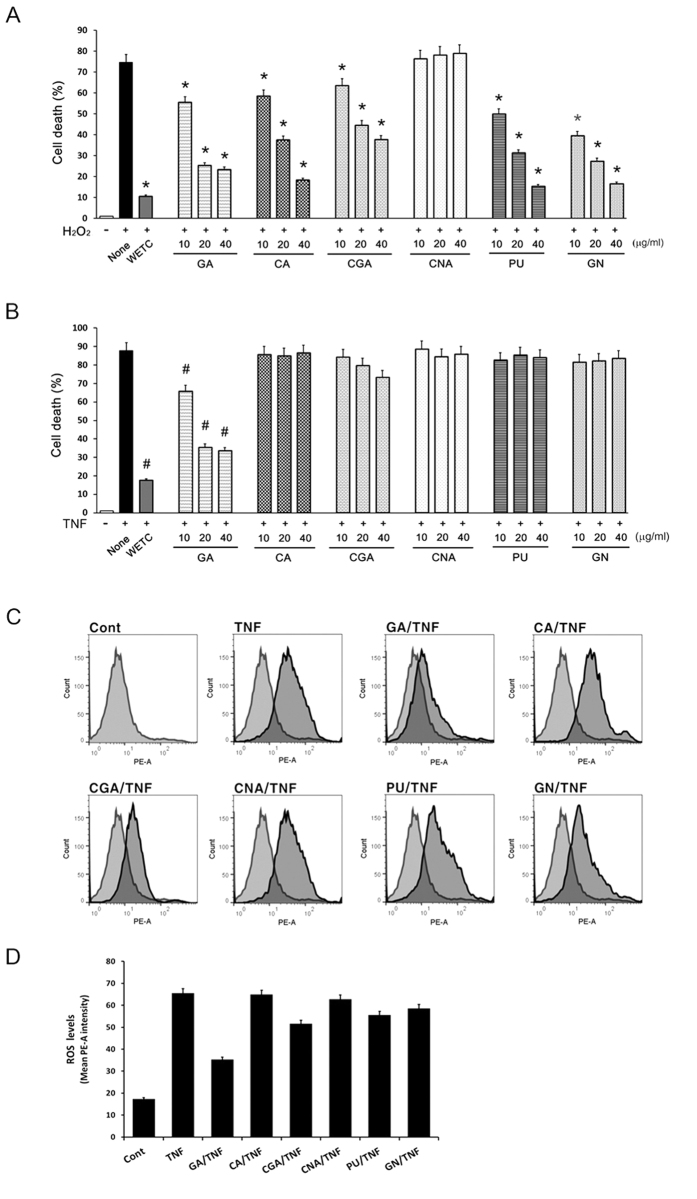
Inhibitory effects of the constituents of WETC on TNF-induced necroptotic cell death and ROS production. (**A**,**B**) L929 cells were pretreated with WETC or indicated constituents for 30 min, followed by H2O2 ((**A**), 500 μM); or TNF ((**B**), 15 ng/mL) for another 8 h. Cell death was quantified as in Fig. 2A. Data represent the mean ± SE of three independent experiments. *P < *0.05*, compared with H2O2-treated group. ^#^*P* < *0.05*, compared with TNF-treated group. (**C,D**) L929 cells were treated with TNF (15 ng/mL) in the absence or presence of indicated compounds for 6 h. The levels of intracellular ROS were monitored as in Fig. 2E. Representative flow cytometry data (**C**). Quantification of ROS from three independent experiments (**D**). GA: gallic acid, CA: chebulic acid, CGA: chebulagic acid, CNA: chebulinic acid, PU: punicalagin, GN: geraniin.

To identify the major constituents responsible for the protective effects of WETC against DR-mediated necroptotic cell death, we compared the protective efficiency of each constituent against TNF- or H_2_O_2_-induced cell death. All of the constituents except chebulinic acid significantly protected L929 cells from H_2_O_2_-induced cell death (Fig. 4A), confirming that those constituents had antioxidant activity, as reported earlier^27^,^28^,^33^,^34^. However, only gallic acid significantly protected against cell death induced by TNF or the combination of TNF, CHX, and z-VAD-FMK in L929 cells or MEFs, respectively. As the protective mechanism of WETC against TNF-induced necroptotic cell death involves decreased ROS production, we subsequently examined the effects of these constituents on the level of intracellular ROS. Gallic acid demonstrated the highest potency for inhibiting TNF-induced ROS production (Fig. 4C,D, Supplementary Fig. 3E). However, its protective effects against TNF-induced necroptotic cell death was weaker than that observed with WETC (Fig. 4B). Therefore, although gallic acid is likely the major contributor to the protection against DR-mediated necroptosis by decreasing TNF-induced ROS production, other unidentified constituents may contribute to the anti-necroptotic effect of WETC.

### Caspase-dependent suppression of DR-mediated apoptosis by WETC and its constituent, punicalagin

**Figure 5:**
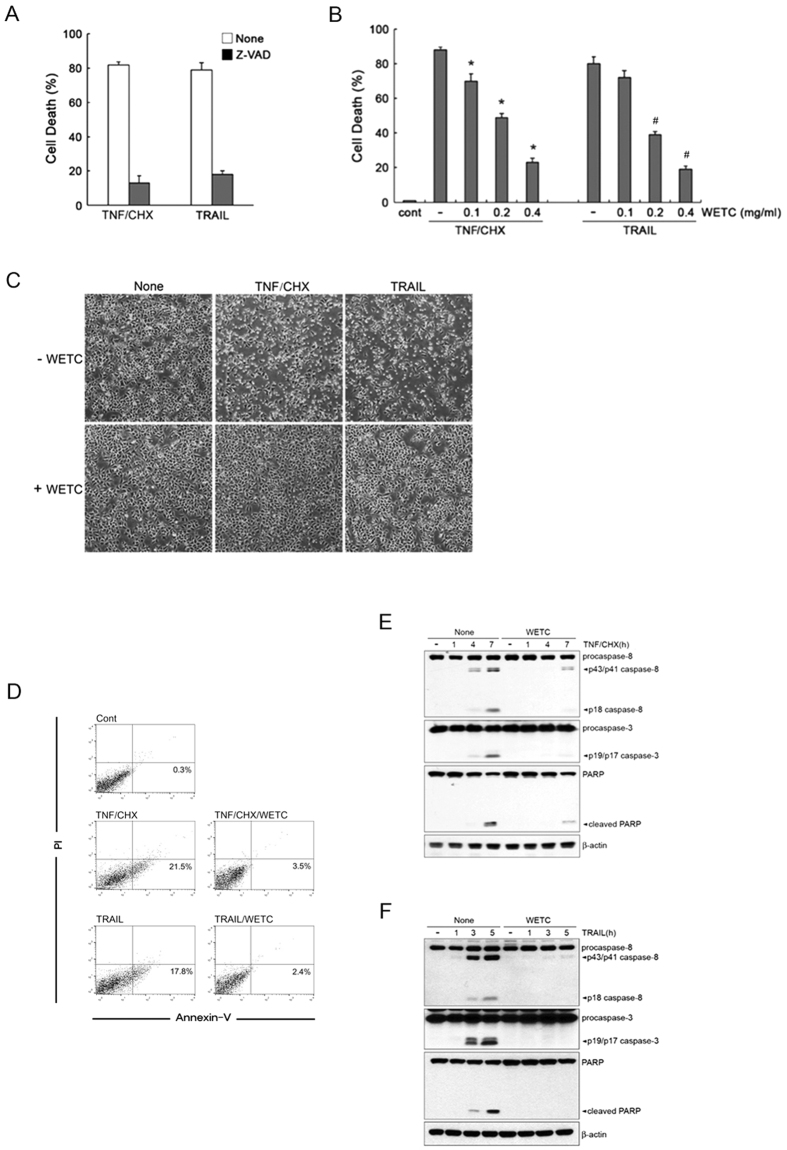
WETC protects DR-mediated apoptotic cell death in response to TNF and TRAIL. (**A**) HeLa cells were pretreated with z-VAD-FMK (20 μM), and then treated with TNF (15 ng/mL) plus CHX (10 μg/mL) or TRAIL (500 ng/mL) for 8 h. Cell death was quantified as in Fig. 2A. (**B**) HeLa cells were pretreated with indicated concentrations of WETC for 30 min, followed by TNF/CHX or TRAIL, and cell death was quantified as in (**A**). *P < *0.05*, compared with TNF/CHX-treated group. ^#^*P* < *0.05*, compared with TRAIL-treated group. (**C,D**) HeLa cells were treated with TNF/CHX or TRAIL in the absence or presence of WETC (0.4 mg/mL) for 8 h. Cell were visualized using a normal inverted microscope (**C**) and the mode of cell death (**D**) was assessed as in Fig. 2B (**E,F**). After pretreatment with WETC (0.4 mg/mL) for 30 min, HeLa cells were treated with TNF plus CHX or TRAIL for indicated times. Whole cell lysates were separated by SDS-PAGE and the immunoblotting was performed with indicated antibodies.

Ligation of DR can cause both apoptotic and necroptotic (nonapoptotic) cell death, suggesting the possibility of crosstalk between these two cell death pathways^7^,^8^,^9^,^10^. Having shown that WETC possesses antagonizing potential against TNF-induced necroptosis, we investigated the effects of WETC on the apoptotic mode of cell death using HeLa cells that preferentially undergo apoptotic cell death after TNF treatment^30^. Pretreatment with z-VAD-FMK significantly abrogated cell death in response to TNF/CHX and TRAIL (Fig. 5A), confirming the occurrence of caspase-dependent apoptotic cell death in these cells. Unexpectedly, pretreatment of WETC dramatically suppressed TNF/CHX- and TRAIL-induced cell death (Fig. 5B,C) and the early phase of apoptosis, as evidenced by the decreased annexin V^+^ population (Fig. 5D, Supplementary Fig. 5). To understand the anti-apoptotic mechanisms of WETC, we examined whether WETC affects the activation of the caspase cascade, including activation of caspase-8 and caspase-3, and the resultant cleavage of poly ADP-ribose polymerase (PARP). WETC pretreatment significantly inhibited TNF/CHX- and TRAIL-induced processing of caspase-8 and the resultant cleavage of caspase-3 and PARP (Fig. 5E,F). These results indicate that WETC efficiently protects not only against DR-mediated necroptotic cell death but also has potent anti-apoptotic function.

**Figure 6:**
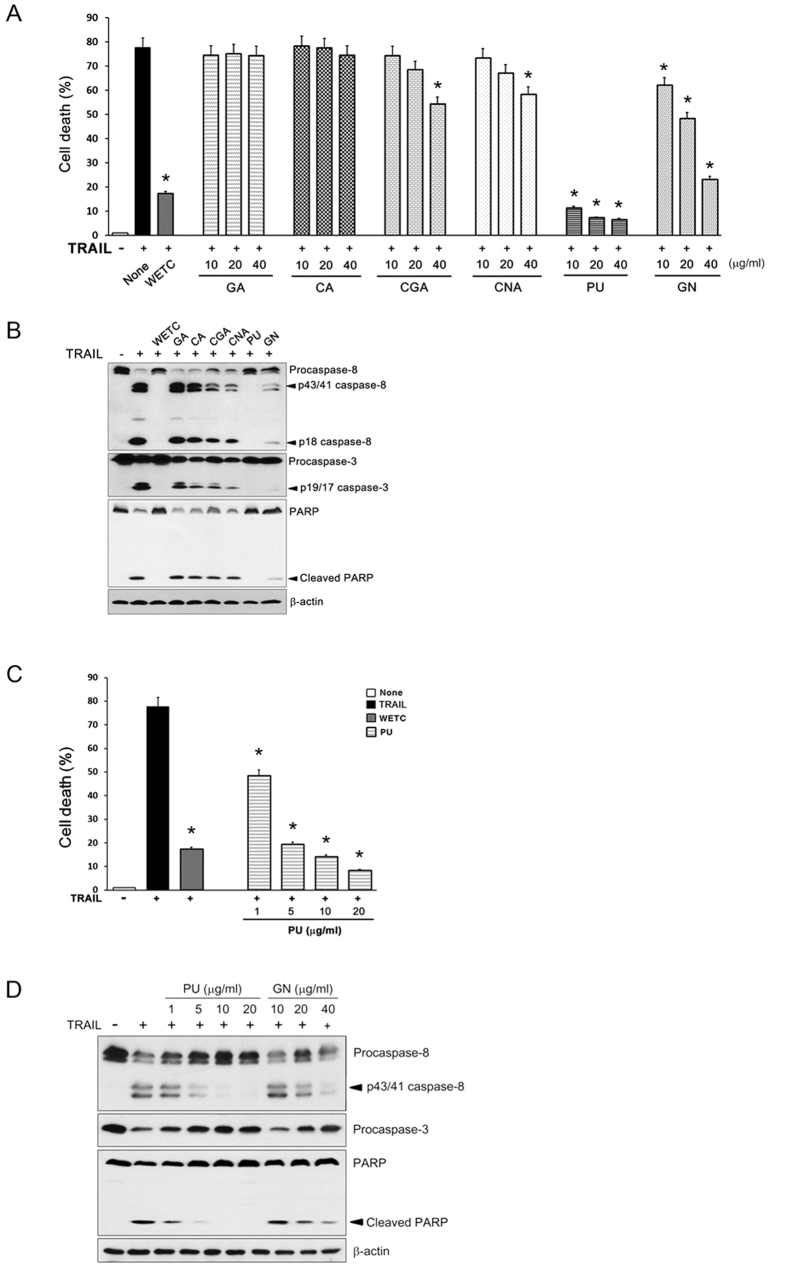
Inhibitory effects of the constituents of WETC on TRAIL-induced apoptotic cell death. (**A**) HeLa cells were pretreated with WETC or indicated constituents for 30 min, followed by TRAIL (500 ng/mL) for another 8 h, and cell death was quantified as in Fig. 2A. *P < *0.05*, compared with TRAIL-treated group. (**B**) After pretreatment with WETC (0.4 mg/mL) or each WETC constituent (40 μg/mL) for 30 min, HeLa cells were treated with TRAIL (500 ng/mL) for another 8 h. Whole cell lysates were separated by SDS-PAGE and the immunoblotting was performed with indicated antibodies. (**C**) HeLa cells were pretreated with WETC (0.4 mg/mL) or indicated concentrations of punicalagin (PU) for 30 min, followed by TRAIL (500 ng/mL) for another 8 h, and cell death was quantified as in (**A**). *P < *0.05*, compared with TRAIL-treated group. (**D**) HeLa cells were pretreated with indicated concentrations of punicalagin (PU) or geraniin (GN) for 30 min, followed by TRAIL (500 ng/mL) for another 8 h. Whole cell lysates were immunoblotted with indicated antibodies as in (**B**).

The antagonizing potential of the WETC constituents against TRAIL-induced apoptotic cell death was determined. Punicalagin and geraniin showed a strong protective effect, whereas chebulagic acid and chebulinic acid showed relatively low protective efficacy, as judged by a cell viability assay (Fig. 6A) and the extent of caspase-8, caspase-3, and PARP cleavages by immunoblot assays (Fig. 6B). Notably, punicalagin pretreatment alone almost completely inhibited TRAIL-induced apoptosis, even to a higher extent than that observed with WETC pretreatment. Caspase-dependent anti-apoptotic potency of punicalagin was observed at a concentration of 1 μg/mL, and complete inhibition occurred at 5 μg/mL; the inhibitory effect of geraniin occurred at 40 μg/mL (Fig. 6C,D). It is worth noting that the concentrations of punicalagin and geraniin were ~4.5 and 30.7 μg/mL, respectively, in a 0.4 mg/mL solution of WETC (Table 1), which exhibited a strong anti-apoptotic effect. Therefore, it is possible that punicalagin is the main constituent responsible for the anti-apoptotic activity of WETC.

### WETC induces robust autophagic flux via inhibiting the mTOR pathway

**Figure 7:**
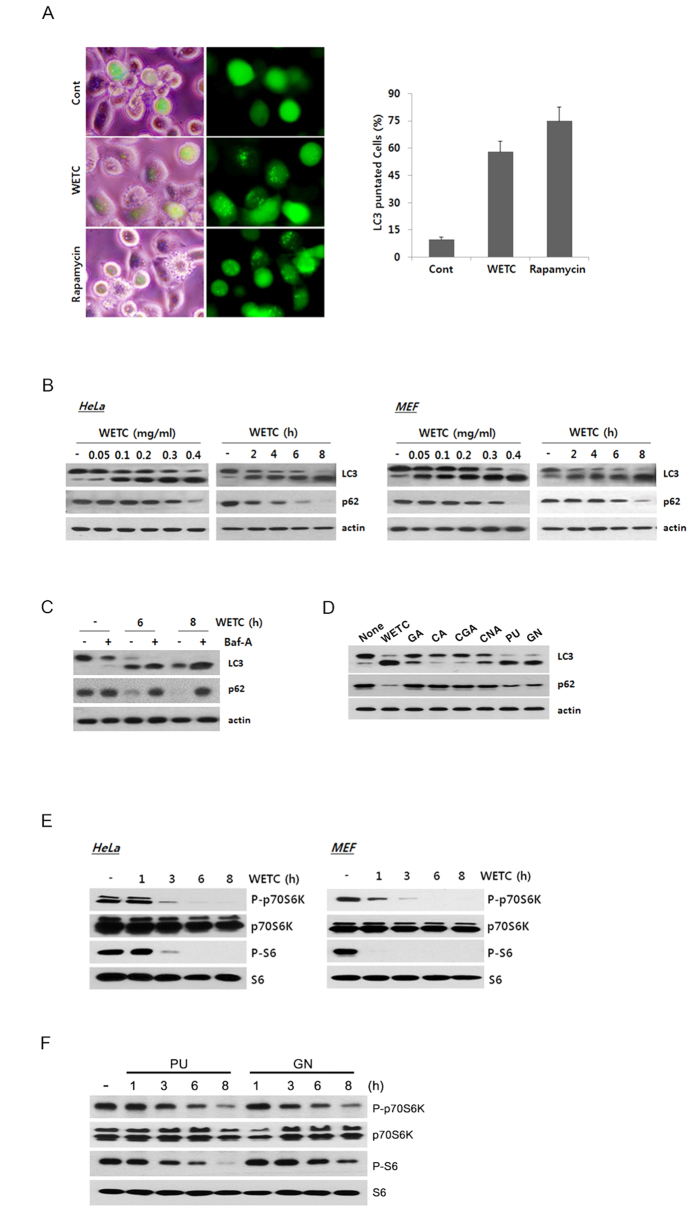
WETC and its constituents (punicalagin and geraniin) induces autophagic flux via mTOR pathway. (**A**) HeLa cells were infected with recombinant adeno-viral expressing GFP-LC3 (100 PFU/cells) for 2 h, and then replaced with DMEM medium. After 24 h, cells were treated with WETC (0.4 mg/mL) and rapamycin (100 nM) for 6 h. Left panel, representative fluorescent images of the cells under a fluorescence microscope. Right panel, the percentage of cells with GFP-LC3 localized to punctated structures was calculated by counting a minimum of 100 cells per sample with values representing the means of triplicate experiments. (**B**) HeLa cells and MEFs were treated with the indicated concentrations (left panels) or times (right panels) of WETC. (**C**) WETC increases autophagic flux. After 30 min pretreatment with bafilomycin A (Baf-A, 100 nM), HeLa cells were further treated with WETC (0.4 mg/mL) for indicated times. (**D**) HeLa cells were treated with WETC (0.4 mg/mL) or each WETC constituent (40 μg/mL) for 6h. (**E**) HeLa cells and MEFs were treated with WETC for indicated times. (**F**) HeLa cells were treated with punicalagin (PU, 5 μg/mL) or geraniin (GN, 40 μg/mL) for indicated times. Whole cell lysates were separated by SDS-PAGE and the immunoblotting was performed with indicated antibodies.

Autophagy and apoptosis are extensively interconnected by various molecular crosstalk mechanisms^35^,^36^,^37^. In many settings, autophagy blocks the induction of apoptosis and inhibits the activation of apoptotic caspases^38^,^39^,^40^. To explore the mechanism underlying the anti-apoptotic function of WETC, we investigated whether WETC provided protection from DR-mediated apoptosis via induction of autophagy. To test this possibility, we infected HeLa cells with recombinant adenoviral vector carrying GFP-tagged LC3 and examined the distribution pattern of LC3, a hallmark of autophagosomes. Untreated cells showed a diffuse pattern of GFP-LC3 distribution, whereas WETC-treated cells showed a high intensity of punctate distribution, representing the increased formation of autophagosomes (Fig. 7A, left panel). GFP-LC3 puncta were quantified in WETC- or rapamycin-treated cells (Fig. 7A, right panel). Furthermore, immunoblotting analysis showed a marked time- and dose-dependent conversion of nonautophagic soluble LC3 (LC3-I) to autophagic LC3 (LC3-II) in response to WETC treatment in multiple cell types, including HeLa cells and MEFs (Fig. 7B, top row). The scaffolding protein p62 is an autophagy receptor that interacts with LC3 and is degraded through the autophagy-lysosome pathway^41^. To verify that WETC enhances the autophagic flux level, we examined the protein level of p62 in the same lysates. Notably, the level of p62 decreased gradually upon WETC treatment, indicating autophagic degradation of p62 (Fig. 7B, second row). To further confirm the promoting effect of WETC on autophagic flux level, we conducted additional autophagic flux analysis using bafilomycin A (Baf-A), an inhibitor of vacuolar ATPase, which blocks acidification of the lysosomes and thereby suppresses lysosomal degradation of proteins within the autophagosome^42^. The addition of Baf-A to WETC-treated cells further enhanced the LC3-II protein level and provided complete protection against p62 degradation (Fig. 7C). Furthermore, punicalagin and geraniin induced a marked conversion of LC3 and degradation of p62, whereas the gallic, chebulic, chebulagic, and chebulinic acids showed no significant effects on the autophagy markers (Fig. 7D). These results confirm that WETC and some of its principle constituents including punicalagin and geraniin trigger autophagosome formation and promote autophagic flux. Moreover, the ability of punicalagin or geraniin to activate autophagy correlates with its inhibition of TRAIL-induced apoptosis in HeLa cells.

Small molecules can induce autophagy activation through either mTOR-dependent or mTOR-independent pathways^43^. Thus, we examined whether WETC-induced autophagy affected the mTOR signaling pathway. Notably, time-dependent analyses indicated that the phosphorylation level of p70S6K, a downstream target of mTORC1, and the phosphorylation level of S6, a downstream target of p70S6K, were markedly reduced at earlier time points (1 to 3 h) after WETC treatment in both HeLa and MEF cells (Fig. 7E). Similarly, treatment of HeLa cells with punicalagin and geraniin led to a time-dependent decrease in the phosphorylation levels of p70S6K and S6 (Fig. 7F). These observations suggest that WETC and its constituents (punicalagin and geraniin) induce autophagy by suppressing the mTORC1 signaling pathway.

### WETC-induced autophagy selectively antagonizes DR-mediated apoptosis

**Figure 8:**
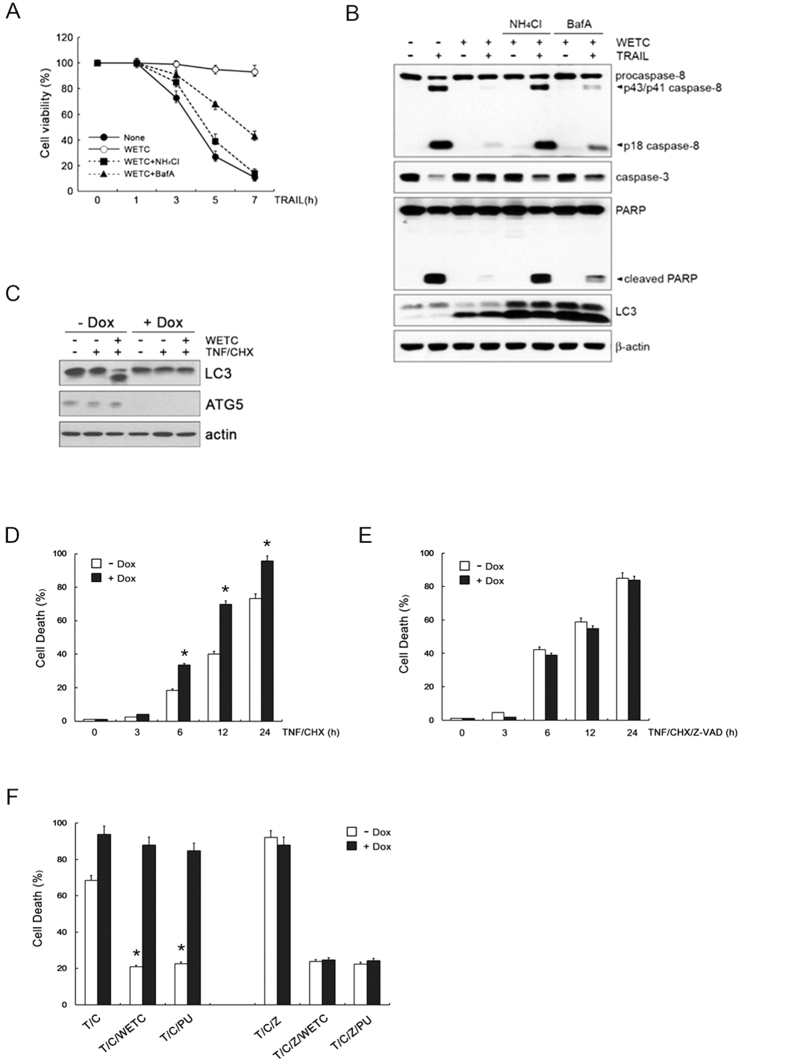
Pharmacological or genetic inhibition of autophagy abolishes the anti-apoptotic potential of WETC. (**A**) HeLa cells were pretreated with autophagy inhibitor, NH_4_Cl (5 mM) and Baf-A (100 nM) for 30 min, and then followed by 500 ng/mL of TRAIL in the absence or presence of WETC (0.4 mg/mL) as indicated times. Cell death was quantified as in Fig. 2A. (**B**) HeLa cells were pretreated with NH_4_Cl and Baf-A, and then followed by TRAIL in the absence or presence of WETC for 6 h. Whole cell lysates were separated by SDS-PAGE and the immunoblotting was performed with indicated antibodies. (**C**) Tet-off Atg5 MEFs were incubated with or without 10 ng/mL doxycyclin hydrochloride (Dox) for 3 d, and then further treated with WETC (0.4 mg/mL) or TNF (15 ng/mL) plus CHX (10 μg/mL) for 6 h. Whole cell lysates were immunoblotted with indicated antibodies. (**D**,**E**) Tet-off Atg5 MEFs were treated with TNF plus CHX (T/C) or z-VAD-FMK, TNF and CHX (T/C/Z) in the absence or presence of Dox (10 ng/mL). (**F**) Tet-off Atg5 MEFs were pretreated with WETC (0.4 mg/mL) or punicalagin (PU, 5 μg/mL) for 30 min, and then followed by T/C or T/C/Z treatment for 18 h. The percentage of cells death was quantified as in *A*. Data represent the mean ± SE of three independent experiments. *P < *0.05*, compared with TNF plus CHX-treated group.

Accumulating evidence suggests that the apoptotic response can be profoundly affected by the ability of autophagome formation or autolysosomal activity^35^,^36^,^37^. In this way, the autophagic processes could limit or delay the apoptotic response upon DR ligation^39^,^44^,^45^. To investigate the function of autophagy, we evaluated the impact of pharmacological autophagy inhibitors on the anti-apoptotic potential of WETC against DR-mediated cytotoxicity. When the cells were pretreated with NH_4_Cl or Baf-A, which inhibit autophagic flux, WETC failed to provide protection against TRAIL-induced apoptotic cell death and activation of the caspase cascade (Fig. 8A,B). These findings support the notion that autophagic activation contributes to the anti-apoptotic activity of WETC upon DR ligation.

To further dissect the effects of WETC-induced autophagy on apoptosis and necroptosis upon DR ligation, we compared the extent of TNF-induced apoptosis and necroptosis after genetically blocking autophagy using the conditional knockout system of Tet-off Atg5 MEFs. This system monitors protein levels following doxycyclin (Dox) treatment (Fig. 8C). Consistently, Atg5 deletion in Dox-treated cells led to obvious TNF-induced apoptotic cell death compared to the control cells (Fig. 8D). In contrast, in the presence of z-VAD-FMK, the extent of TNF-induced necroptic cell death in Dox-treated cells was similar to that of control cells (Fig. 8E), indicating that autophagy activation acts to limit apoptosis rather than necroptosis upon TNFR1 ligation. In parallel, we further dissected the effects of autophagy on the anti-apoptotic and anti-necroptotic activities of WETC in Tet-off Atg5 cells. Deletion of Atg5 following Dox treatment led to abrogation of anti-apoptotic activity of WETC and punicalagin in TNF/CHX treated cells (Fig. 8F, left panel). Notably, anti-necroptotic activity of WETC and punicalagin in TNF/CHX/z-VAD treated cells was not affected by Atg5 deletion (Fig. 8F, right panel), indicating that WETC or punicalagin-induced autophagy selectively antagonized apoptosis, but not necroptosis. These data support our hypothesis that WETC or punicalagin induces autophagy via the mTOR-dependent pathway, which limits DR-mediated apoptosis.

## Discussion

*T*. *chebula* and its constituents possesses cytoprotective activity against various oxidative stress-mediated pathological conditions such as brain ischemia, renal reperfusion injury, gastric ulcer, burn wounds, and drug-induced hepatotoxicity^16^,^46^,^47^,^48^,^49^. Based on earlier pharmacological and biochemical studies, this plant exhibits antioxidant and free radical-scavenging activities *in vitro* and *in vivo*^18^,^19^. In view of these findings, the large body of research regarding the cytoprotective effects of *T*. *chebula* has focused predominantly on necrotic cell death^30^,^32^. As ROS have a crucial role in TNF-induced necroptotic cell death, it is assumed that *T*. *chebula* may inhibit necrotic cell death. In accordance with previous observations under conditions of oxidative stress, we found that WETC antagonizes mitochondrial-derived ROS production and thereby inhibits TNF-induced necroptotic cell death. These results constitute direct evidence that the antioxidant properties of WETC may modulate necroptotic cell death induced after ligation of DRs, such as TNFR1. Based on the fact that several constituents of *T*. *chebula*, such as gallic acid, chebulic acid, punicalagin, and geraniin, have shown antioxidant potential, we thus hypothesized that these constituents may contribute to the anti-necroptotic function of WETC upon DR ligation. As expected, most of them (punicalagin, geraniin, and gallic, chebulic, and chebulagic acids) offered significant protection against H_2_O_2_-induced cell death. Gallic acid efficiently suppressed necroptosis and ROS production in TNF-treated cells whereas the others showed little effect on TNF-induced ROS accumulation, suggesting that these may target different sources of ROS production (mitochondria-derived ROS production versus exogenous ROS accumulation). Hence, further study is needed in this area. We also found that co-treatment of cells with gallic acid showed no synergistic protective effects with other constituents against TNF-induced necroptotic cell death (data not shown), suggesting that other unidentified constituents, in addition to gallic acid, may act cooperatively in suppressing TNF-induced ROS production that leads to necroptotic cell death.

Although previous reports have described the chemopreventive efficacy of WETC against ROS-mediated necrotic cell death^18^,^19^, it has remained unclear whether WETC also modulates apoptotic cell death. In this study, we demonstrated for the first time that, in addition to anti-necroptotic properties, WETC provides efficient protection against the apoptotic mode of cell death in response to TNF or TRAIL. As ROS seem to be dispensable in the DR-mediated apoptotic signaling pathway, the antioxidant properties of WETC are unlikely to be involved in the anti-apoptotic mechanism. One important finding from this study is that WETC, or rather its constituents such as punicalagin and geraniin can promote autophagic flux via the mTORC1 signaling pathway. In terms of function, there is abundant evidence indicating that a certain level of autophagy has an important housekeeping role in the degradation of cytoplasmic components in response to stress conditions, thereby promoting survival^50^. Accordingly, some autophagy core machinery genes are upregulated in TNF-resistant cells^45^. Furthermore, it has been reported that autophagy functions as a cytoprotective signal against TRAIL-induced apoptosis^39^,^51^. In agreement with these reports, we found that the protective capacity of WETC against TNF- or TRAIL-induced apoptotic, but not necroptotic cell death, was almost completely abrogated under autophagy blockade conditions with pharmacological inhibitors or Atg5 deletion. These findings indicate that autophagy activation by WETC modulates the apoptotic signaling pathway exclusively under DR ligation conditions. While these *in vitro* data are promising, quantitative pharmacokinetic studies of WETC or its constituents in *in vivo* models are critical to develop future clinical applications. In this regard, no human intervention studies including pharmacokinetic parameters, absorption and metabolism have been conducted with *T*. *chebula* extract. However, in a previous study, punicalagin was detected at a peak concentration of 17.5 μg/mL in an obese rat model following oral administration of pomegranate extract containing punicalagin^52^. In addition, punicalagin has been shown to prevent mitochondrial loss and ameliorate oxidative stress via AMPK activation in the same experimental model^53^. Thus, it is possible that punicalagin could circulate throughout the body, inducing beneficial effects. In this study, punicalagin was demonstrated to be a critical component that accounted for the anti-apoptotic potential of WETC via suppression of the mTOR signaling pathway. One important hint from these observations is that the concentration of punicalagin (1–5 μg/mL) that exhibited strong efficacy on apoptotic cell death or autophagy activation was about 3.5 or 17.5 times lower than that in the serum of the experimental rat model^52^, suggesting that such concentrations of punicalagin may be clinically relevant. However, to the best of our knowledge, no quantitative pharmacokinetic analyses using the *T*. *chebula* extract *in vivo* have been reported. Further bioavailability and metabolic profile studies of WETC standardized to punicalagin using *in vivo* preclinical models or human trials are required to address this important issue.

It remains unclear how such autophagy protects cells against DR-mediated apoptosis. It has been reported that autophagy can sequestrate the pro-form of caspage-8 into autolysosomes in TRAIL-resistant cells, thereby leading to the degradation of active casapse-8^54^, which is required for DR-mediated caspase-dependent apoptosis. However, we observed no autophagic degradation of procaspase-8 upon WETC treatment (data not shown), suggesting that the inhibitory function of autophagy on DR-mediated apoptosis is unlikely to be achieved through caspase-8 degradation. Having shown that the transcriptional regulation of autophagy genes plays an essential role in the execution phase of autophagy and in the acquisition of resistance to DR-mediated apoptosis, future functional proteomics and molecular network analyses are required to fully understand the precise mechanisms through which WETC modulates interplay between autophagy and apoptotic machineries.

In summary, we identified the mechanisms by which WETC protects against dual modes of cell death (necroptosis and apoptosis) upon DR ligation; first, WETC suppresses TNF-induced necroptosis by antagonizing mitochondrial-driven ROS production and, second, WETC blunts DR-induced apoptosis via promotion of autophagy through suppression of mTORC1. Our study presents the novel hypothesis that *T*. *chebula* might show effectiveness against diseases characterized by aberrantly sensitized apoptotic or non-apoptotic signaling cascades.

## Methods

### Chemicals and reagents

All commercial antibodies and chemicals were purchased from the following resources: Anti-caspase-3 and anti-caspase-9 antibodies (Cell Signaling Technology); anti-poly(ADP-ribose) polymerase(PARP) antibody (BD Biosciences Pharmingen); anti-LC3B, anti-Atg5 and anti-actin antibodies, chebulagic acid, punicalagin, chebulinic acid, gallic acid, 3-methylaednine (3-MA), wortmanin (WM), bafilomycin A1 (BafA1), doxycyclin and cycloheximide (Sigma-Aldrich); chebulinic acid (ChemFaces); geraniin (Dalian Meilun Biotech Co., Ltd, China); the pancaspase inhibitor Z-VAD-FMK, the caspase-8 inhibitor z-IETD-FMK and the caspase-3 inhibitor z-DEVD-FMK (Calbiochem); Mito-Tempo [(2-(2,2,6,6 tetramethylpiperidin-1-oxyl-4-ylamino)-2-oxoethyl) triphenylphosphonium chloride, monohydrate] (ALX-430–150-M005) (Enzo Life Sciences); Recombinant mouse TNF-α and TRAIL (R&D Systems).

### WETC preparation

For the initial screening of natural products, the dried pellets of a plant extract library of traditional oriental medicine were purchased from the Plant Diversity Research Center (Daejeon, Korea). These pellets were resuspended in distilled water at 20 mg/mL and used for the initial screening. Dried fruits of *T. chebula* were purchased from an herbal pharmaceutical company (Jeong-Seong Drugstore, Daejeon, Rep. of Korea) and herb identification was confirmed by Professor SI Yim (a herbology specialist at Oriental Medical College, Daejeon University). A total 100 g *T. chebula* was ground and boiled in 1 L distilled water for 90 min using an automatic non-pressure pot (Dae-Woong, Seoul, Korea). The extract was centrifuged for 15 min at 150 × *g*, and the supernatant was lyophilized using a vacuum freeze drying system and stored at −20 °C. The extraction yield was 11.58% (w/w).

### Chemical composition and quantitative analysis of WETC

The chemical components of WETC were identified using an Agilent 1100 series high-performance liquid chromatography (HPLC) system (Agilent Technologies, Santa Clara, CA, United States). Samples (10 μL in 90% methanol/water) were injected into the HPLC connected to a Gemini C18 column (5 μm; 250 × 4.6 mm; Phenomenex, Torrance, CA, United States) at room temperature. Data were acquired with ChemStation software (version A 10.01). The column was eluted at a flow rate of 0.4 mL/min using 0.1% formic acid in acetonitrile (solvent A) and water (solvent B). The following gradient program was employed: 0–5 min, linear gradient from 100 to 95% B; 5–40 min, linear gradient from 95 to 60% B. Eluting compounds were detected at 276 nm. For quantification of WETC, pure reference compounds (punicalagin, geraniin, and gallic chebulic, chebulagic, and chebulinic acids) were dissolved in methanol/water (9:1) at a range of concentrations between 12.5 and 1000 μg/mL to generate a standard curve from the corresponding peak areas. The samples were analyzed under identical conditions, and the concentrations of each compound in WETC were determined by extrapolating from the standard curve. To confirm the chemical compositions of WETC, mass spectrometric analysis was performed using an ultra-high-performance liquid chromatography high-resolution mass spectrometry (UHPLC-HRMS). For analysis, 5 mg WETC dissolved in 1 mL 90% methanol solution were analyzed on an LTQ Orbitrap XL linear ion-trap MS system (Thermo Scientific Co., San Jose, CA) equipped with an electrospray ionization source. The separation was performed on an Accela UHPLC system using an Acquity BEH C18 column (1.7 μm; 100 × 2.1 mm; Waters, Milford, MA, USA). The mobile phase conditions contained 0.1% formic acid in acetonitrile (solvent A) and water (solvent B). The gradient flow was as follows: 0–1 min, isocratic with 99% B; 1–7 min, linear gradient from 99 to 10% B; 7–10 min, isocratic with 10% B. The flow rate was 0.4 mL/min, and the full-scan mass spectra were recorded in the negative ionization scan mode between *m/z* 150 and 1,500. An Orbitrap analyzer was used for high-resolution mass data acquisition with resolving power of 30,000 FWHM at *m/z* 400.

### Cell culture

L929 cells, HeLa cells, and doxycylin-inducible Atg5 null MEF cells were cultured in Dulbecco’s modified Eagle’s medium supplemented with 2mM glutamine, antibiotics (100 U/mL penicillin G and 100 ug/mL streptomycin), and 10% heat-inactivated FBS, and maintained in a humidified incubator at 37 °C in 5% CO2 atmosphere.

### Immunoblot analysis and immunoprecipitation

Cells were grown and treated with WETC or its constituents in 60-mm tissue culture plates. The cells were collected and lysed in M2 buffer (20 mM Tris at pH 7.6, 0.5% NP-40, 250 mM NaCl, 3 mM EDTA, 3 mM EGTA, 2 mM DTT, 0.5 mM PMSF, 20 mM 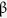-glycerol phosphate, 1 mM sodium vanadate, 1 ug/mL leupeptin). Protein concentration was determined using Bio-Rad protein assay reagent. 20 μg of the cell lysates were fractionated by SDS-polyacrylamide gel electrophoresis (PAGE) and blotted onto PVDF membrane. After blocking with 5% skim milk in PBS/T, the membrane was proved with the relevant antibody and visualized by enhanced chemiluminescence (ECL), according to the manufacturer’s instruction (Amersham). For immunoprecipitation assays, the lysates were mixed and precipitated with the relevant antibodies and protein G-A agarose beads by overnight incubation at 4 °C. The beads were washed three times with M2 buffer, and the bound proteins were resolved in 10% SDS-PAGE.

### Determination of cell death

Cell death was quantified using trypan blue exclusion assay as described previously^30^. Briefly, cells were washed, trypsinzed and mixed with trypan blue dye for 5 min. The number of stained cells (dead cells) were counted by hemacytometer, and expressed as a percentage of total cells. For measurement of early apoptotic or necroptotic cell death, cells were stained for 10 min at room temperature with 10 μM fluorescein isothiocyanate (FITC)-labeled annexin V (BD Biosciences PharMingen), and propidium iodide (PI; BD Biosciences PharMingen), in a Ca^2+^-enriched binding buffer (10 mM HEPES, pH 7.4, 140 mM NaCl, and 2.5 mM CaCl2), and analyzed by two-color flow cytometry. Annexin V and PI emissions were detected in the FL1 and FL2 channels of a FACSCalibur flow cytometer (BD Biosciences), using emission filters of 488 and 532 nm, respectively. The annexinV^−^/PI^−^ population was regarded as normal healthy cells, whereas annexinV^+^/PI^−^ cells were taken as a measure of early apoptosis and annexinV^+^/PI^+^ as necrosis/late apoptosis

### Determination of intracellular ROS production

Production of intracellular ROS was measured using the fluorescent dye 2,7-dichlorofluorescein diacetate (DCF-DA), which readily diffuse intro cells where it is hydrolyzed to 27-dichlorofluorescein, a nonfluorescent polar compound that is trapped within cells. Cells were then washed and stained with 5 μM CM-H2DCFDA (Calbiochem) in Hank’s balanced salt solution (HBSS) for 30 min before collecting cells. The stained cells were analyzed with a FACSCalibur flow cytometer, and data were processed with CellQuest software (BD Bioscience).

### Immunofluorescence analysis

To detect GFP-LC3 translocation, HeLa cells were grown on glass coverslips and then infected with Ad-GFP-LC3 (100 viral particles/cells). After 24 h, cells were treated with WETC or rapamycin for 6 h and fixed with 4% paraformaldehyde. Cells were permeabilized with PBS containing 0.2% Triton X-100 and 0.1 M glycine. The cells were then washed twice with PBS. Translocation of GFP-LC3 from cytosol to autophagic vacuoles were imaged under a confocal laser-scanning microscope (LSM5 Live, ZEISS). Quantitative analysis of LC3 fluorescence images was measured with the software LSM Image Browser (version 3.5, ZEISS).

### Statistical analysis

Data are expressed as the mean ± S.E. from at least three separate experiments performed triplicate. The differences between groups were analyzed using a Student’s t test, and *P* < 0.05 is considered statistically significant. Statistical analyses were carried out using SPSS software (ver. 11.0; SPSS Inc., Chicago, IL, USA).

## Additional Information

**How to cite this article**: Lee, Y. *et al.* *Terminalia Chebula* provides protection against dual modes of necroptotic and apoptotic cell death upon death receptor ligation. *Sci. Rep.* **6**, 25094; doi: 10.1038/srep25094 (2016).

## Supplementary Material

Supplementary Information

## Figures and Tables

**Table 1:** Quantitative analytical results of WETC constituents.

Compounds	Retention time (min)	Chemical formula	Molecular weight	Contents (mean ± SD, nmole/mg)
Gallic acid	10.59	C_7_H_6_O_5_	170.12	553.79 ± 3.76
Punicalagin	18.47	C_48_H_28_O_30_	1084.71	10.48 ± 0.21
Geraniin	20.17	C_41_H_28_O_27_	952.64	80.78 ± 0.40
Chebulic acid	23.34	C_14_H_12_O_11_	356.23	54.60 ± 0.62
Chebulagic acid	25.56	C_41_H_30_O_27_	954.66	9.24 ± 0.08
Chebulinic acid	29.10	C_41_H_32_O_27_	956.67	10.32 ± 0.47
