# Supplementary material for: Terminalia Chebula provides protection against dual modes of necroptotic and apoptotic cell death upon death receptor ligation
Source: Sci Rep. 2016 Apr 27;6:25094. doi: 10.1038/srep25094 (PMC4846877; doi:10.1038/srep25094)
Supplement: Supplementary Information [file srep25094-s1.pdf]

***Terminalia Chebula* provides protection against dual modes of necroptotic and apoptotic cell death upon death receptor ligation**

Yoonjung Lee[1,#], Hee Sun Byun[1,#], Jeong Ho Seok[1,#], Kyeong Ah Park[1], Minho Won[1], Wonhyoung Seo[1], So-Ra Lee[1], Kidong Kang[1], Kyung-Cheol Sohn[2], Ill Young Lee[3], Hyeong-Geug Kim[4], Chang Gue Son[4], Han-Ming Shen[5], Gang Min Hur[1*]

Supplementary Figure 1. Structure of six constituents in *Terminalia Chebula* extract

Supplementary Figure 2. Calibration curves of six reference compounds. The constituents of WETC were serially diluted at the indicated concentration ranges. HPLC analysis was performed with a Gemini C18 column at 276 nm to calculate each peak area. Insets represent the regression equation (y = ax + b) with the correlation coefficient (r^2).

Supplementary Figure 3. Dot plot analysis using forward and side scatter light parameters (FSC *versus* SSC) under TNF-induced necroptotic conditions in L929 cells and MEFs. (A) L929 cells were pretreated with a pancaspase inhibitor (z-VAD-FMK, 20 μM), a necroptosis inhibitor (Nec-1, necrostatin-1, 10 μM) or WETC (0.4 mg/mL), followed by TNF (15 ng/mL) for another 8 h. (B) MEFs were pretreated with Nec-1 (10 μM) or WETC (0.4 mg/mL) for 30 min, and then treated with z-VAD-FMK (20 μM), TNF (15 ng/mL) and cycloheximide (CHX, 10 μg/mL) for 18 h. (C) L929 cells were treated with TNF (15 ng/mL) for indicated times. (D) L929 cells were pretreated with WETC (0.4 mg/mL) or mitochondria-targeted antioxidant Mito-TEMPO (100 μM) for 30 min,

followed by TNF (15 ng/mL) for another 6 h. (E) L929 cells were treated with TNF (15 ng/mL) in the absence or presence of the WETC constituents for 6 h. The population and distribution of cells were analyzed in the scatter plot of FSC vs. SSC by flow cytometry.

Supplementary Figure 4. Inhibitory effects of the constituents of WETC on TNF-induced necroptotic cell death in MEFs. MEFs were pretreated with WETC or indicated WETC constituents for 30 min, followed by z-VAD-FMK (20 μM), TNF (15 ng/mL) and cycloheximide (CHX, 10 μg/mL) for 18 h. Cells were trypsinized and collected in PBS, and cell death was quantified by trypan blue exclusion assay. Data represent the mean ± SE of three independent experiments. *, $P < 0.05$, compared with TNF/CHX/z-VAD-FMK-treated group.

Supplementary Figure 5. Dot plot analysis using forward and side scatter light parameters (FSC *versus* SSC) under DR-mediated apoptotic conditions in HeLa cells. HeLa cells were treated with TNF (15 ng/mL) plus CHX (10 μg/mL) or TRAIL (500 ng/mL) in the absence or presence of WETC (0.4 mg/mL) for 8 h. The population and distribution of cells were analyzed in the scatter plot of FSC vs. SSC by flow cytometry.

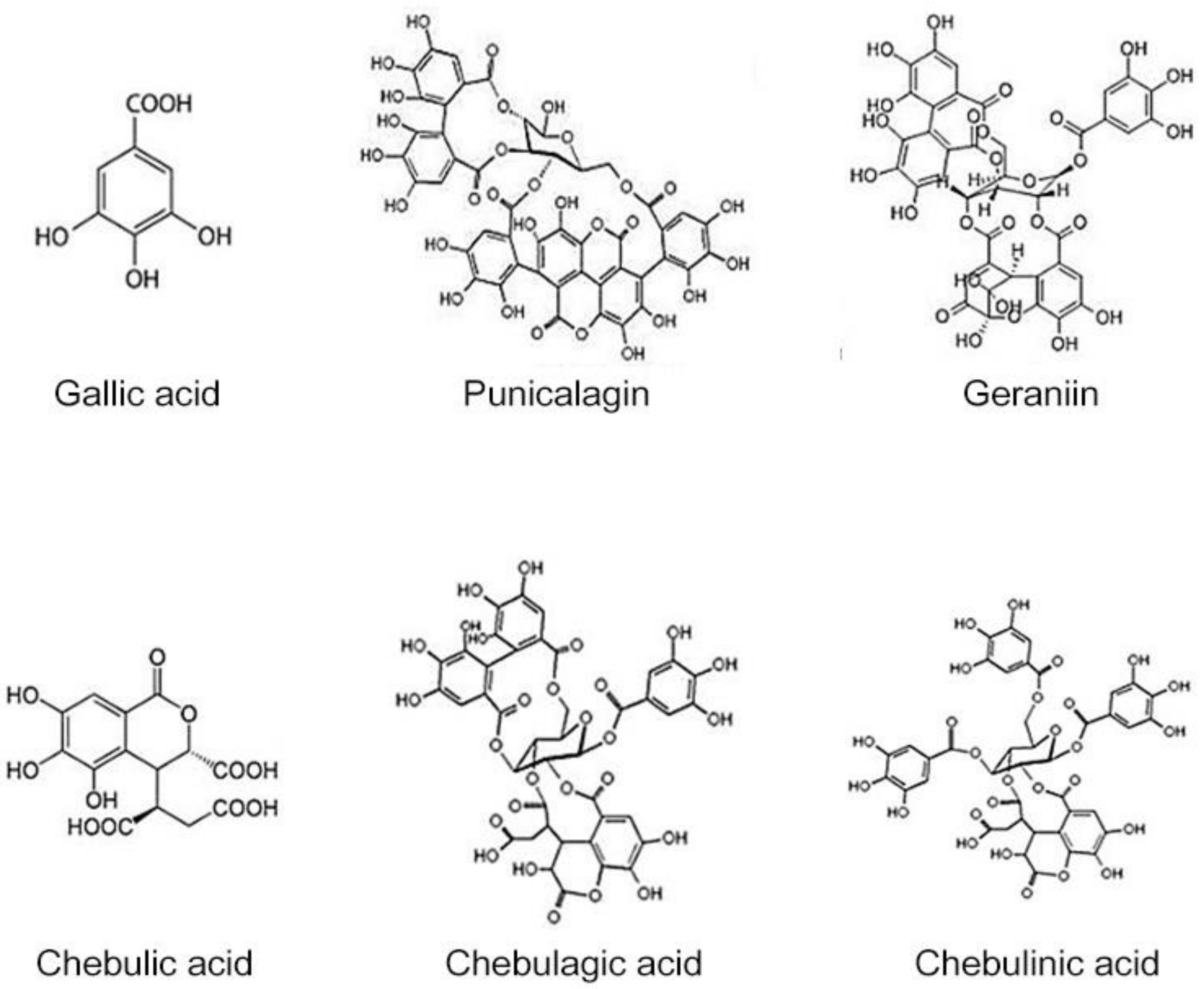

Supplementary Figure 1. Structure of six constituents in *Terminalia Chebula* extract

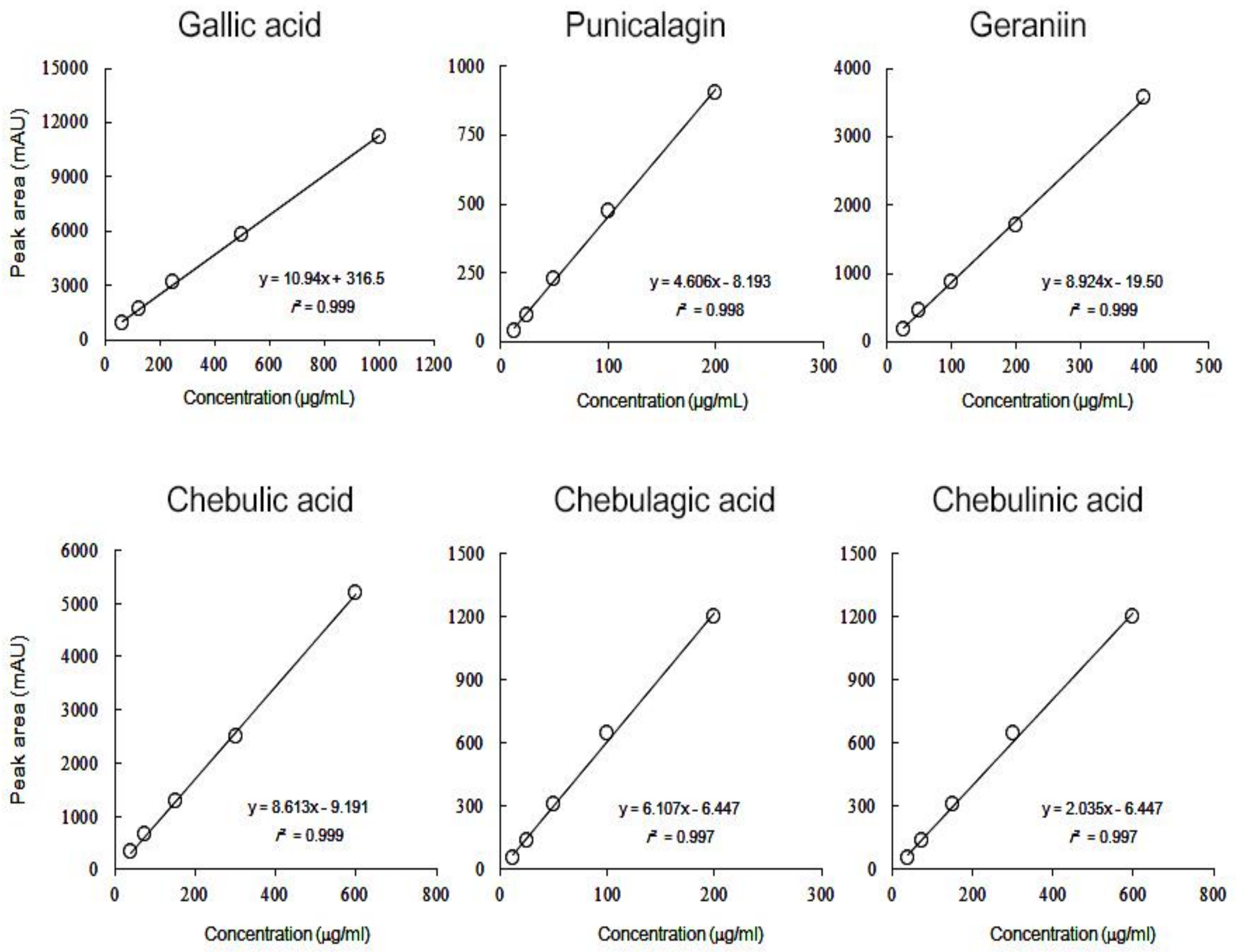

Supplementary Figure 2. Calibration curves of six reference compounds. The constituents of WETC were serially diluted at the indicated concentration ranges. HPLC analysis was performed with a Gemini C18 column at 276 nm to calculate each peak area. Insets represent the regression equation (y = ax + b) with the correlation coefficient (r^2).

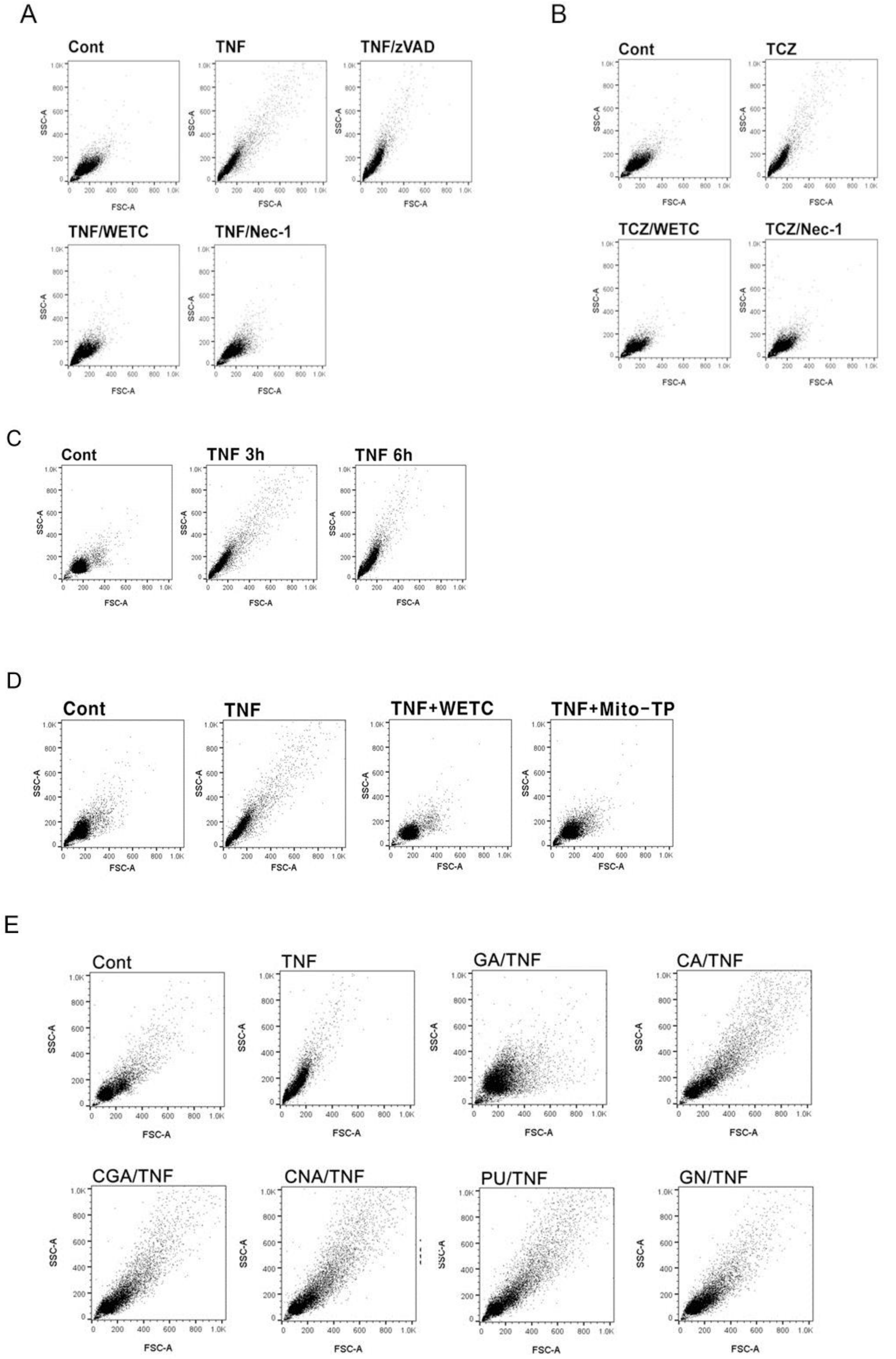

Supplementary Figure 3. Dot plot analysis using forward and side scatter light parameters (FSC *versus* SSC) under TNF-induced necroptotic conditions in L929 cells and MEFs. (A) L929 cells were pretreated with a pancaspase inhibitor (z-VAD-FMK, 20 μM), a necroptosis inhibitor (Nec-1, necrostatin-1, 10 μM) or WETC (0.4 mg/mL), followed by TNF (15 ng/mL) for another 8 h. (B) MEFs were pretreated with Nec-1 (10 μM) or WETC (0.4 mg/mL) for 30 min, and then treated with z-VAD-FMK (20 μM), TNF (15 ng/mL) and cycloheximide (CHX, 10 μg/mL) for 18 h. (C) L929 cells were treated with TNF (15 ng/mL) for indicated times. (D) L929 cells were pretreated with WETC (0.4 mg/mL) or mitochondria-targeted antioxidant Mito-TEMPO (100 μM) for 30 min, followed by TNF (15 ng/mL) for another 6 h. (E) L929 cells were treated with TNF (15 ng/mL) in the absence or presence of the WETC constituents for 6 h. The population and distribution of cells were analyzed in the scatter plot of FSC vs. SSC by flow cytometry.

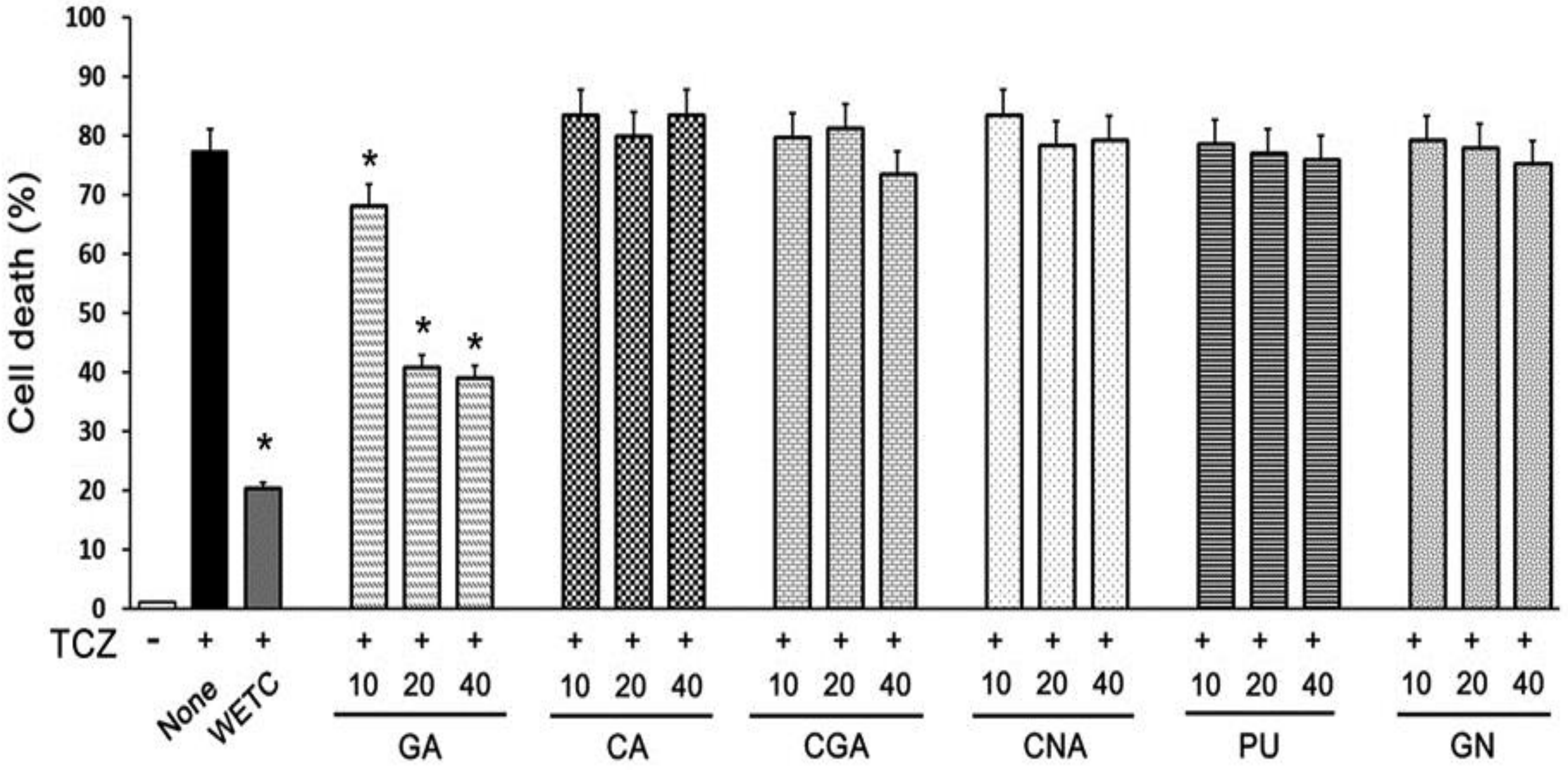

Supplementary Figure 4. Inhibitory effects of the constituents of WETC on TNF-induced necroptotic cell death in MEFs. MEFs were pretreated with WETC or indicated WETC constituents for 30 min, followed by z-VAD-FMK (20 μM), TNF (15 ng/mL) and cycloheximide (CHX, 10 μg/mL) for 18 h. Cells were trypsinized and collected in PBS, and cell death was quantified by trypan blue exclusion assay. Data represent the mean ± SE of three independent experiments. *, $P < 0.05$, compared with TNF/CHX/z-VAD-FMK-treated group.

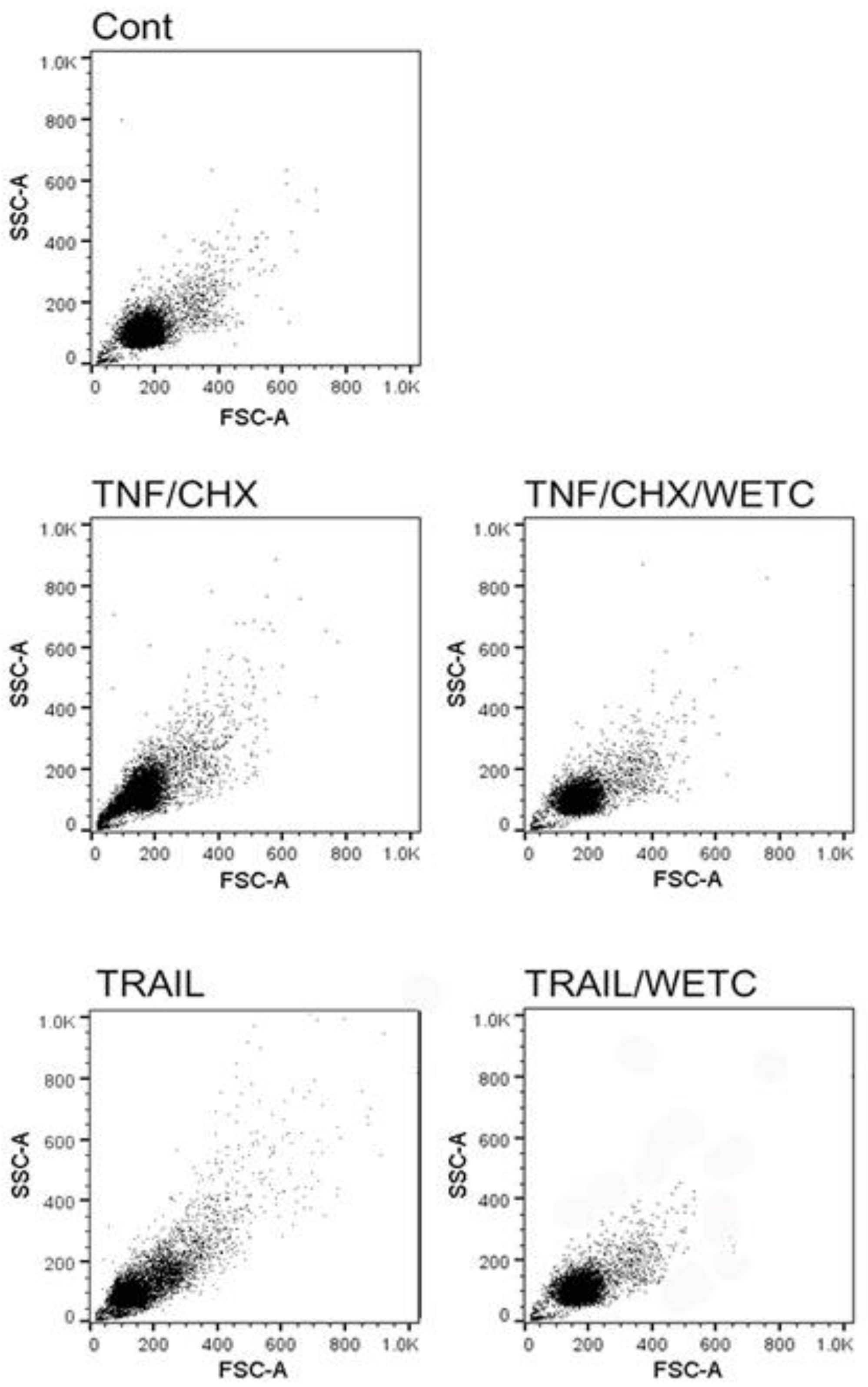

Supplementary Figure 5. Dot plot analysis using forward and side scatter light parameters (FSC *versus* SSC) under DR-mediated apoptotic conditions in HeLa cells. HeLa cells were treated with TNF (15 ng/mL) plus CHX (10 μg/mL) or TRAIL (500 ng/mL) in the absence or presence of WETC (0.4 mg/mL) for 8 h. The population and distribution of cells were analyzed in the scatter plot of FSC vs. SSC by flow cytometry.
